# Enhancing Rabbit Welfare in Intensive Production Systems: A Review

**DOI:** 10.1155/tswj/1056471

**Published:** 2026-07-31

**Authors:** Iryna Lastovska, Tetyana Dyman, Andriy Getya, Oleksandr V. Borshch, Mykhailo Matvieiev, Oleksandr O. Borshch, Svitlana Polishchuk, Thobela Louis Tyasi

**Affiliations:** ^1^ Department of Technology Milk and Meat Production, Bila Tserkva National Agrarian University, Bila Tserkva, Ukraine; ^2^ Department of Applied Biology, Animal Breeding and Genetic, National University of Life and Environmental Science of Ukraine, Kyiv, Ukraine; ^3^ Department of Animal Technology, National University of Life and Environmental Science of Ukraine, Kyiv, Ukraine; ^4^ Department of Agricultural Economics and Animal Production, University of Limpopo, Polokwane, Limpopo, South Africa, ul.ac.za

**Keywords:** behavioral indicators, cage systems, disease prevention, environmental enrichment, industrial rabbit farming, physiological indicators, rabbit welfare, sustainable agriculture

## Abstract

Rabbit farming is an important sector of animal husbandry worldwide, providing meat, fur, and research models. However, intensive production systems often compromise the welfare of rabbits by restricting their natural behaviors and exposing them to physical and psychological stressors. This article reviews key factors affecting the welfare of farmed rabbits in industrial settings, emphasizing the ethical and sustainable dimensions of modern animal husbandry. It discusses housing systems, including traditional cages, enclosures, and free‐range models, highlighting their impacts on rabbit health and behavior. The role of environmental enrichment, appropriate nutrition, disease prevention, and biosecurity in promoting rabbit well‐being is explored. Furthermore, the article examines contemporary welfare assessment methods and the potential of precision livestock farming technologies for continuous monitoring and individualized care. Despite challenges related to economic and infrastructural constraints, integrating welfare‐centered practices, staff training, and regulatory measures is critical for enhancing the quality of life for farmed rabbits, improving productivity, and meeting consumer demands for ethical and sustainable production.

## 1. Introduction

Rabbit farming constitutes a significant sector of animal husbandry in numerous countries [1]. Rabbits are valued for their high‐quality meat [2], fur [3], and are extensively utilized in scientific research [4]. The growing demand for rabbit products has driven a shift toward intensive farming systems, which often neglect animal welfare considerations [5]. Between 2008 and 2018, the global population of rabbits raised for meat production increased by 9.8% [6]. In recent decades, animal welfare has become a critical scientific and ethical concern. Traditional husbandry practices, which primarily emphasize productivity, frequently overlook the ethological and physiological needs of rabbits; nonetheless, housing conditions directly affect animal health, behavior, and the quality of products [7].

Economic efficiency in production must not come at the expense of physical and psychological well‐being [8, 9]. Consequently, establishing, implementing, and adhering to welfare standards is essential to ensuring survival and comfortable housing. Consumer awareness regarding animal welfare has grown markedly, as reflected in European initiatives such as the European Citizens′ Initiative “End the Cage Age,” advocating the phase‐out of cages for all farmed animals by 2027 [10]. Rabbits are particularly sensitive to environmental conditions, including noise, lighting, ventilation, and space, and their social nature requires opportunities for species‐specific behaviors such as digging, jumping, and social interaction [11]. The World Organisation for Animal Health [12, 13] has developed a set of animal welfare recommendations that are recognized in international trade. Although no specific standard for rabbits currently exists, the principles of the “five freedoms” and “fitness for purpose on the farm” are applicable across all species. The European Union is a global leader in implementing animal welfare regulations, including those concerning rabbits. In 2017, the European Parliament adopted a resolution calling for a gradual transition from conventional cages to alternative housing systems such as pens or enclosures. The resolution recommends a minimum space allocation of at least 0.5 m^2^ per rabbit, the provision of elevated platforms for jumping, shelters and bedding for digging, and group housing for adult animals [14].

In parallel, research efforts [15–18] are addressing the impact of housing conditions on rabbit productivity, health, and welfare, including the use of intelligent monitoring technologies. These findings provide a scientific basis for future national recommendations and standards. This article is aimed at analyzing the main factors affecting rabbit welfare in industrial farming systems, identifying challenges in current housing practices, reviewing international welfare standards, and proposing practical recommendations to improve the well‐being of farmed rabbits.

## 2. Factors Affecting the Welfare of Farmed Rabbits

Animal welfare is a multidimensional concept that encompasses physical health, psychological comfort, the ability to express natural behavior, and the absence of stress, pain, and suffering. The definition of welfare was formulated by the World Organisation for Animal Health (OIE) [12] and is based on the concept of the “five freedoms” proposed in 1965 by the British Farm Animal Welfare Council (FAWC). According to this concept, animals should have freedom from hunger and thirst—access to clean water and adequate food; freedom from discomfort—provision of appropriate shelter and conditions for rest; freedom from pain, injury, and disease—through prevention and timely treatment; freedom to express normal behavior—through sufficient space, appropriate equipment, and social contact; freedom from fear and distress—through conditions that minimize psychological stress.

**Figure 1:**
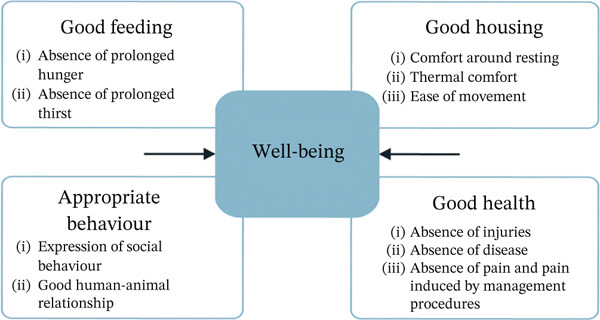
Welfare principles and criteria according to Welfare Quality.

These points have become the basis for the modern assessment of the welfare of farm animals, including rabbits. Although the concept of the five freedoms [19] is somewhat simplified, it provides a practical basis for monitoring and improving housing conditions. Almost all studies are related to the analysis of welfare assessment results over a certain period of time, and the Welfare Quality (EFSA) project is aimed at improving animal welfare through standard welfare assessment metrology and translate these assessments into understandable information (Figure 1).

In recent years, there has been a growing trend in the scientific community toward a broader understanding of welfare—not only as the absence of suffering, but as comfortable living throughout life. This includes the opportunity for animals to experience pleasure, play, explore their environment, and form social bonds. In this context, increasing attention is being paid to environmental enrichment and the emotional state of animals [20]. Rabbits, as a species, have distinct behavioral needs, the neglect of which in intensive production conditions can lead to stereotypical behavior, aggression, self‐injury, and reduced productivity. That is why understanding and applying welfare principles is critically important not only for ethical but also for economic reasons [21]. The welfare of rabbits in farm conditions largely depends on a complex of factors, including both the physical environment and social and behavioral aspects. Rabbits are traditionally kept in cages, which often do not meet their physiological and behavioral needs. Insufficient space limits their ability to jump, stand at full height, dig, or hide, leading to stress and the development of abnormal behavior [20]. Ideal conditions require not only adequate space, but also a platform for jumping, shelter, and bedding that allows them to dig and keep warm. The main systems for keeping rabbits—in multi‐tier cages, enclosures, and free‐range—have a number of limitations (Table 1).

**Table 1:** Welfare‐oriented assessment of rabbit housing systems.

Housing system	Key features	Space per animal	Behavioral opportunities	Welfare implications	Advantages/limitations, +/−
Multi‐tier cage systems	Galvanized mesh cages, individual confinement, mesh flooring	~0.3 m^2^	Very limited: jumping, running, rearing, digging restricted	High stress, stereotypies, pododermatitis, skeletal abnormalities; social behaviors prevented	+ Hygienic, easy to manage
					− Restrictive, chronic stress, poor musculoskeletal development
Enclosure systems	Solid floor with dense bedding; group housing; stable social groups	≥ 0.75 m^2^	Moderate to high: running, jumping, digging, social interaction	Supports natural behaviors, reduces stress, improves bone and muscle development; lowers pododermatitis risk	+ Promotes welfare and social behavior
					− Requires careful hygiene, risk of aggression if groups improperly formed
Free‐range systems (pasture farming)	Access to fenced pastures; burrowing; natural foraging	Variable, typically ≥ 5 m^2^ per animal	Very high: free movement, burrowing, foraging, choice of food, exploration	Optimal welfare, supports full behavioral repertoire, improves digestion and dental health	+ Closest to natural conditions
					− Predator risk, parasite control, weather protection needed; mainly suitable for small or organic farms

## 3. Housing of Rabbits

*Multi-tier cage systems* are a traditional system for keeping rabbits, especially on an industrial scale. It is based on the use of multi‐tier cages, usually made of galvanized mesh. The standard dimensions of such cages (often 50 × 60 × 40 cm) significantly restrict the natural behavior of animals. Rabbits are deprived of the opportunity to perform basic motor activities: make more than 1–2 jumps in a row, run, stand on their hind legs at full height (observation pose), and dig. According to the European Food Safety Authority (EFSA) [20], the inability to stretch out and stand on their hind legs is a significant stress factor and can lead to skeletal abnormalities [22].

*Enclosure systems* are a progressive alternative to cages and involve keeping socially stable groups of rabbits on a solid floor with dense bedding (straw and sawdust). A sufficient enclosure area (recommended to be at least 0.75 m^2^ per animal) allows rabbits to move freely, run, jump, and deep bedding allows them to satisfy their need to dig. The availability of space contributes to the normal development of the muscular and skeletal systems [23].

*Free-range systems* (pasture farming) are closest to the natural conditions of the wild ancestors of rabbits. It provides animals with access to fenced pastures where they can graze freely. The animals can fully satisfy their behavioral needs—they can not only move around freely and dig burrows, but also choose their own food (various grasses), which has a positive effect on their digestive system and dental health [24]. Regardless of the housing system, appropriate feeding strategies, thermal comfort, and proper microclimatic control are essential prerequisites for maintaining both health and productivity [20]. A complete diet is the basis for well‐being. Rabbits need a high‐fiber diet, including hay, to support gastrointestinal function and tooth wear. Lack of fiber or water can cause serious illness [25]. Automatic waterers are often used on large farms, but it is important to monitor their functionality [26, 27].

### 3.1. Environmental Factors Affecting Rabbits Welfare

Temperature, humidity, ventilation, and lighting directly affect the health of rabbits. The optimal temperature is 15°C–20°C. High temperatures cause heat stress, and excessive humidity leads to the development of respiratory diseases. Incorrect lighting can disrupt the animals′ biorhythms [28]. Importantly, the growing recognition of animal welfare not only as the absence of suffering but also as the provision of opportunities for positive experiences underscores the need for continuous refinement of rabbit husbandry practices.

## 4. A Comprehensive Approach to the Prevention of Diseases in Rabbits in Industrial Farming Conditions

The intensification of rabbit production, characterized by high animal density, short production cycles, and technological stress, creates favorable conditions for the emergence and rapid spread of diseases of various etiologies. The incidence of diseases and mortality, particularly among young rabbits during the postweaning period, can be substantial, resulting in significant economic losses for rabbit farms. An effective prevention system is not just a set of therapeutic measures, but a comprehensive strategy that encompasses biosecurity, management, feed optimization, and specific immunoprophylaxis [29]. The purpose of this analysis is to systematize modern scientific approaches to the prevention of rabbit diseases in industrial conditions [30]. Successful prevention is impossible without understanding the main risk factors inherent in intensive systems. Excessive animal density per unit area is a catalyst for the transmission of pathogens. In addition, it creates social stress, which leads to an increase in the level of cortisol in the blood of animals [31]. It has been scientifically proven that chronic stress suppresses the immune system, making animals more vulnerable to opportunistic infections such as pasteurellosis and intestinal diseases caused by *Clostridium perfringens*.

Ventilation problems lead to the accumulation of ammonia (NH_3_) and carbon dioxide (CO_2_) in the room, which irritate the mucous membranes of the respiratory tract and create conditions for the development of respiratory diseases. Temperature fluctuations and high humidity are also significant stress factors, especially for young animals [32]. Inadequate cleaning and disinfection of premises and equipment between production cycles leads to the accumulation of infectious pressure. Fecal contamination of feed and water is the main route of transmission of coccidiosis and other intestinal infections. Diets that are not balanced in terms of fiber, protein, and carbohydrate content are the root cause of most digestive disorders. A deficiency of indigestible fiber is particularly dangerous, leading to a slowdown in gastrointestinal motility and an imbalance of microorganisms in the cecum [33]. Specific prevention is aimed at preventing the most dangerous and economically significant infections, primarily viral hemorrhagic disease of rabbits (RHDV), myxomatosis, pasteurellosis, and coccidiosis (Table 2). The “all empty/all occupied” system is critically important. After the premises are vacated, thorough mechanical cleaning and disinfection with preparations effective against oocysts (e.g., based on cresol or ammonia) is carried out. Mesh flooring in cages reduces contact with feces but has negative consequences for animal welfare (pododermatitis). Digestive disorders are the leading cause of mortality in young rabbits in industrial rabbit farming. Most of them are noninfectious or polyetiological in nature and related to feeding.

**Table 2:** Key rabbit diseases, their preventive measures, and welfare impact.

Disease	Causative agent	Transmission/risk factors	Clinical signs	Prevention/control	Degree of welfare impact	References
Rabbit hemorrhagic disease (RHDV1, RHDV2)	Calicivirus (RHDV1, RHDV2)	Direct contact, contaminated feed, water, fomites; RHDV2 affects rabbits from 15 to 20 days	Peracute mortality up to 90%–100%	Vaccination with inactivated bivalent vaccines; primary immunization at 4–6 weeks, revaccination as recommended; immunize breeding stock to ensure colostral immunity	Severe— sudden mass mortality	Abrantes et al. [34]; Capucci et al. [35]; Dalton et al. [36]
Myxomatosis	Myxoma virus (poxvirus)	Bites of blood‐sucking insects (mosquitoes, fleas), direct contact	Edema (myxomas) on head and body	Combined vaccination with RHDV; mosquito nets on windows/vents; elimination of stagnant water; disinsection	Severe— high morbidity, chronic suffering	Kerr and Best [37]; Kerr [38]
Pasteurellosis (infectious rhinitis, “contagious runny nose”)	*Pasteurella multocida* (bacterium)	Endogenous pathogen activated by stress, poor ventilation, overcrowding	Rhinitis, pneumonia, abscesses	Improve microclimate (ventilation, reduce ammonia), avoid overcrowding and stress; commercial or autogenous vaccines (reduce symptoms but do not eliminate carriers)	Moderate—chronic respiratory distress	Deeb and DiGiacomo [39]; Abd El‐Ghany [40]
Coccidiosis	*Eimeria* spp. (protozoa)	Fecal–oral transmission; common in young animals postweaning	Intestinal or hepatic forms; diarrhea, poor growth	Use coccidiostats (robenidine, diclazuril, salinomycin) in feed during risk period; rotate drugs to prevent resistance	Moderate—reduced growth, welfare impairment	Pakandl [41]

**Figure 2:**
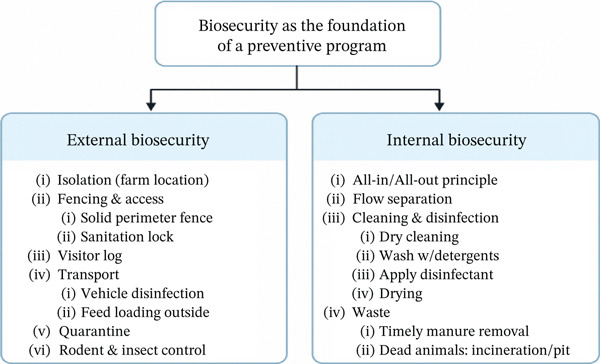
Biosecurity as the cornerstone of preventive health programs.

Postweaning enteropathy (epizootic rabbit enteropathy [ERE]) is a complex syndrome characterized by diarrhea, bloating, and high mortality. Its development is associated with a combination of factors: weaning stress, feed change, and immaturity of the digestive system. Diet plays a key role in disease prevention. Scientific research, in particular the work of [42], proves that fiber is the basis of a rabbit′s gastrointestinal health. The diet should contain high levels of indigestible fiber (lignin and cellulose) and moderate levels of digestible fiber (hemicellulose and pectin). The acid derergent fiber content should not be less than 18%–20% [43]. An insufficient amount of coarse fiber slows down peristalsis, leading to stagnation of contents in the cecum and the development of dysbiosis—excessive growth of pathogenic bacteria (*Clostridium* spp.*, Escherichia coli*) [44]. The content of easily digestible carbohydrates (starch) in the diet for young animals should be limited (to 10%–12%), as its excess leads to acidosis in the cecum and disruption of cecotrophy. Limited feeding (70%–80% of ad libitum consumption) is practiced in the first 2–3 weeks after weaning, which reduces the load on the gastrointestinal tract. Concentrate feed supplements are important in rabbit diets for providing essential nutrients that support growth, reproduction, and productivity, especially in intensive systems. However, excessive use can disrupt digestive balance, reduce fiber intake, and lead to gastrointestinal issues, highlighting the need for careful balance with adequate fiber to maintain rabbit health and welfare [20, 43]. It is important to ensure constant access to clean water and high‐quality hay. Thus, the intensification of rabbit production significantly increases the risk of infectious and noninfectious diseases due to high stocking density, microclimatic imbalances, inadequate hygiene, and nutritional deficiencies. Effective prevention requires a holistic strategy that integrates biosecurity, environmental and feeding management, and targeted immunoprophylaxis. Vaccination against RHDV and myxomatosis, strict hygiene protocols, optimized diets rich in indigestible fiber, and careful postweaning management are key components of disease control. Ultimately, sustainable rabbit production depends on preventing rather than treating diseases, with emphasis on reducing stressors, maintaining optimal husbandry conditions, and ensuring balanced nutrition. The biosecurity (biosafety) scheme as the foundation of the preventive program is shown in Figure 2.

## 5. Ensuring Rabbit Welfare in Intensive Production Systems: Behavioral, Environmental, and Health Perspectives

Rabbit behavior represents a complex set of actions influenced by both genetic factors and the conditions in which animals are kept. Understanding their behavioral needs is essential to ensuring welfare across various settings, including industrial production, laboratory research, and pet ownership. Cage size is a critical determinant of rabbits′ ability to express natural behaviors. Studies indicate that restricted space significantly limits activities such as jumping, running, and environmental exploration. Scientists [21, 45, 46] reported that rabbits housed in small cages or pens spend more time resting and less time exploring their surroundings. In contrast, provision of larger enclosures promotes activity and environmental interaction. Notably, a recent study in *Animal Welfare* (2023) [47] found that rabbits in large pens were significantly more likely to run, jump, and hop compared with those in smaller enclosures, emphasizing the importance of sufficient space to meet their physiological needs for movement. Social behavior is a fundamental aspect of rabbit life, as they naturally form groups in the wild. Isolation can lead to undesirable behaviors, including stereotypies. A UK animal shelter study revealed that solitary rabbits were more likely to gnaw on cage bars than those housed in pairs (*Animal Welfare*, 2023) [47]. Pair housing also facilitated a quicker return to normal behavior following stressors such as handling. However, some research found no significant differences in aggression between rabbits from different litters in large pens versus rabbits from the same litter in small cages (*Animal Welfare*, 2023) [47]. Overall, opportunities for social interaction—particularly for young rabbits—are considered crucial for psychological well‐being.

Environmental enrichment provides rabbits with diverse stimuli and objects to express natural behaviors, including digging, gnawing, hiding, and exploring. A study [21] demonstrated that providing sticks for gnawing reduced stereotypical behaviors such as cage‐bar chewing. Other examples of environmental enrichment for rabbits include offering untreated wooden blocks, fruit tree branches, cardboard tubes, tunnels, shelters, and bedding for digging. These enrichments increase feeding and drinking time, stimulate exploration, and reduce abnormal behaviors [48]. Elevated platforms have also been shown to positively influence behavior [49]. Together, these findings highlight the need not only for sufficient space but also for opportunities to actively interact with the environment. Although rabbits are inherently social, group housing requires careful management of aggression, particularly among young females due to protective behavior. Individual and group housing systems each have advantages and limitations that must be considered in farm design [50].

Disease prevention is another critical aspect of welfare. Regular veterinary examinations, vaccination programs, and isolation of sick animals are essential, with particular attention to myxomatosis, viral hemorrhagic disease, and stomatitis [51]. Environmental enrichment also supports the expression of instinctive behaviors and reduces stress. Animals in enriched environments exhibit lower stress levels and higher productivity [16, 52]. Effective enrichment stimulates natural exploratory, manipulative, and feeding behaviors, particularly in confined housing [53]. Objects such as untreated wooden blocks, fruit tree branches, cardboard tubes, and boxes not only prevent boredom but also facilitate natural dental wear [54]. Structural enrichment—including platforms, tunnels, and shelters—expands usable space and allows hiding, an important antistress behavior. Platforms additionally enable observation postures [55]. Food enrichment can involve scattering feed in deep bedding or using dispenser toys, encouraging natural foraging behavior. Hay, as the primary dietary component, should be continuously available to occupy a significant portion of daily activity [56]. Ensuring proper welfare requires a holistic approach; even minor deficiencies in one aspect can substantially impair rabbits′ well‐being. Despite growing awareness of welfare importance, commercial systems often limit the expression of natural behaviors, negatively affecting physiological and psychological health [57].

On most industrial farms, rabbits are confined in small metal cages, preventing jumping, rearing, or environmental exploration. Such conditions contribute to muscle atrophy, osteoporosis, and skeletal disorders [58]. Typical cages also lack shelters, play objects, and digging opportunities, leading to stereotypical behaviors such as bar gnawing and repetitive movements, indicative of chronic stress [59]. Solitary confinement can cause depression due to social deprivation, whereas group housing can provoke aggression, particularly during puberty or under resource limitation. Resulting injuries include wounds, bites, and hair loss [60].

In densely populated facilities with insufficient ventilation and high humidity, infections spread rapidly, posing a serious risk to rabbit welfare. Poor air quality can lead to respiratory issues, weakening their immune systems and making them more susceptible to diseases. High humidity creates an ideal breeding ground for bacteria, fungi, and parasites, which can cause a range of infections such as pasteurellosis, respiratory infections, and skin diseases. Overcrowding exacerbates stress among rabbits, which in turn compromises their ability to fight off infections. Stress also leads to abnormal behaviors such as aggression or self‐mutilation, further degrading their welfare. Inadequate ventilation prevents the removal of ammonia produced by urine, which irritates the respiratory tract and eyes, promoting chronic health conditions. Ensuring proper ventilation systems that maintain clean, dry air and controlling humidity levels are crucial steps for minimizing disease outbreaks. Providing enough space for each rabbit reduces stress and limits direct contact, which helps slow the spread of contagious diseases. Regular cleaning and disinfection of the facility, combined with routine health monitoring, further support a healthy environment [61, 62].

Selective breeding for high productivity often overlooks genetic traits associated with adaptability, which can lead to excessively rapid growth or increased muscle mass that may cause pain, skeletal deformities, and reduced overall welfare [63, 64]. Insufficiently trained personnel further compromise care through delayed disease detection or rough handling [65, 66]. Collectively, these observations indicate that conventional farming systems frequently fail to provide even minimum welfare standards for rabbits. Addressing these challenges requires adopting sustainable husbandry practices that prioritize the behavioral, physiological, and psychological needs of the animals.

### 5.1. Assessing and Ensuring the Welfare of Rabbits in Agricultural Production: Indicators, Technologies, and Practices

To ensure the proper level of rabbit welfare, it is important to have objective methods for assessing it. Modern science offers an integrative approach that includes both animal‐based and resource‐based indicators. These include physiological indicators, behavioral assessment, clinical examination, productivity indicators, environmental indicators, and welfare scale assessment [67].

One of the most reliable methods for assessing animal welfare is the analysis of physiological parameters associated with stress or discomfort. Such indicators include cortisol concentrations in blood or saliva, heart rate, body temperature, and changes in appetite or body weight [19]. Although these measures can provide a precise evaluation of the animal′s condition, their application requires specialized equipment and trained personnel, and the procedures themselves may induce considerable stress in rabbits [68]. Behavior represents an important, accessible, and noninvasive indicator of animal welfare. Observable signs include normal activity, exploration, grooming, and digging; abnormal behavior includes apathy, stereotypies, aggression, and cannibalism; and social interactions include avoidance, dominance, and group aggression [69]. Such classification of animal behaviors provides a structured framework for welfare assessment.

Systematic observation of behavior allows welfare problems to be detected at an early stage, often before the appearance of clinical signs. Physical examination of the animal′s body for injuries, ulcers, scratches, and the condition of the coat, eyes, teeth, paws, and hindquarters provides further insights into hygiene, bedding comfort, and overall health status. For example, paw lesions are commonly associated with wire‐mesh flooring in cages. However, such assessments, as well as determining productivity through weighing and body measurements, may themselves be stress‐inducing for the animals.

Animal welfare has a direct influence on reproductive performance, growth rates, juvenile mortality, and disease incidence. A deterioration in welfare is almost invariably accompanied by a decline in productivity [29]. Assessment of the housing microclimate—including air quality (ammonia and carbon dioxide concentrations), temperature, humidity, lighting, ventilation, flooring type, and cage area—constitutes an integral component of comprehensive diagnostics and represents a noninvasive method [70]. To standardize welfare assessment, European researchers have developed scoring systems such as *Welfare Quality* (2009) [19], which integrate multiple criteria into a composite evaluation. These approaches have been adapted for use in rabbits, particularly under experimental conditions.

Thus, combining diverse methods—from behavioral and clinical indicators to physiological and environmental measures—enables a more comprehensive understanding of the welfare status of rabbits on farms. Such an integrated approach facilitates both the early identification of welfare concerns and the implementation of effective corrective measures. In response to the limitations of conventional cage systems, increasing attention is being directed by farms, researchers, and animal welfare organizations toward alternative housing models. The primary objective is to create conditions that accommodate the biological and behavioral needs of rabbits while maintaining productivity. One such approach is the refinement of cages, including modifications to their dimensions and internal design. Within cage systems, welfare can be enhanced by environmental enrichment measures such as the provision of shelters, wooden blocks for gnawing, tunnels, and bedding for digging. These interventions have been shown to reduce stress and improve the psychological and emotional state of animals.

Another promising approach is enclosure‐based housing, which allows rabbits to move freely, explore their environment, and engage in social interactions. Key advantages include increased space, designated areas for resting, feeding, and digging, opportunities for the expression of social behavior, and a reduction in stereotypies [71]. Challenges include the management of aggression, the need for regular cleaning, and greater infrastructural costs. Properly managed group housing of growing rabbits and adult males can further enhance social welfare. Critical requirements include maintaining stable group composition, providing adequate space, and creating areas that allow seclusion and gradual introduction of new individuals [72]. Group housing has been shown to stimulate physical activity, improve social communication, and reduce anxiety.

Modern rabbit farms are increasingly adopting automated monitoring technologies—including video surveillance and motion, temperature, and humidity sensors—to continuously track animal behavior, welfare, and physiological parameters. These systems not only facilitate day‐to‐day management but also help to prevent critical welfare issues. Finally, the establishment of a strong welfare culture within farms is largely dependent on staff competence. Training in animal handling, early recognition of disease symptoms, and behavioral changes can significantly improve the quality of care. An additional key strategy involves ensuring biosecurity through regular disinfection, restricted access, vaccination, and the quarantine of newly introduced animals [73].

### 5.2. Digital Solutions for Achieving Welfare

Consumer surveys conducted in various countries have demonstrated a growing public interest in issues related to animal welfare [74–76]. For example, according to a survey of Eastern European residents [75], Ukrainian respondents associated animal welfare primarily with slaughter conditions and the humane treatment of animals. They also expressed the belief that the quality of animal‐derived products obtained from animals raised under welfare‐friendly conditions is higher compared with those produced without such standards.

Within the European Union, the majority of rabbits (approximately 66%) are raised on commercial farms [49]. It is evident that large‐scale commercial operations generally exhibit higher stocking densities compared with smallholder or family farms. In response, the concept of *precision livestock farming* (PLF) has gained momentum in recent years. This advanced approach to animal husbandry utilizes continuous, real‐time monitoring of individual animals through the integration of sensor technologies, data analytics, and automated systems, which supports evidence‐based management decisions and contributes to improved animal welfare [77, 78]. The application of PLF typically relies on advanced data analytics, often involving mathematical modeling and algorithmic approaches [79].

However, in rabbit farming, this concept remains at an early stage of development [80]. Notably, machine learning algorithms have recently demonstrated promising results, for instance, by accurately predicting rabbit live weight based on body measurements [81]. A specialized device, eFeederRab, has been developed to record feed intake‐related traits in rabbits using RFID (radio frequency identification) ear tags [82]. To ensure precision feeding in cage‐based systems, a robotic feeder has been proposed [83]. Other smart feeding technologies in rabbit production are reviewed by Goswami et al. [63].

In addition, computer vision–based systems are being designed for multiple applications, including monitoring body weight [84], assessing postoperative pain [85], and classifying rabbit behavior [86]. For evaluating the activity of growing rabbits, accelerometer‐based data have been employed [86], whereas aerial thermal imaging has been used to monitor groups under free‐range conditions [87]. Overall, smart livestock farming systems are receiving growing attention in rabbit production research. A prominent example is the development of the farm management software FarmKeep [88], along with a variety of other digital tools. These systems provide diverse functionalities aimed at improving the efficiency of rabbit farming, including advanced analytics, mobile accessibility, and pedigree management. Table 3 summarizes the key innovations in precision feeding for rabbit farming.

**Table 3:** Technological innovations in rabbit welfare and precision livestock farming.

Aspect	Findings/applications	References
Precision livestock farming (PLF)	Continuous collection of individual animal data via sensors supports welfare‐oriented management. Relies on advanced analytics and algorithmic modeling.	Jiang et al. [77]; Matvieiev et al. [78]; Akkem et al. [79]
Early‐stage application in rabbits	Machine learning predicts live weight from body measurements.	Önder et al. [81]
	Development of eFeederRab device for feed intake traits (RFID ear tags).	Sánchez et al. [82]
	Robotic feeder for precision feeding in cages.	Jiang et al. [83]

Smart feeding technologies	Precise monitoring of individual feed consumption.	Norton and Cambra‐López [80] Goswami et al. [63]
	Precision nutrition techniques that supply the appropriate amount of feed with suitable composition to individual animals or groups (part of the PLF approach): automatic data collection, data processing, and control actions.	Pomar and Remus [89]
	Genetic selection for feed efficiency. Future possibilities for genetic selection in rabbits focus on improving residual feed intake, aiming to reduce feed intake without affecting weight gain.	Gidenne et al. [90]
	Use of nonconventional feed additives (e.g., guava leaf extract) for improving growth performance, feed conversion efficiency, and overall health of rabbits.	Abd El‐Aziz [91]
	Environmental enrichment for alleviation stress in rabbit farming, enhancing animal welfare and potentially improving productivity.	El‐Sabrout et al. [53]

Computer vision systems	Body weight monitoring.	Duan et al. [84]
	Postoperative pain assessment.	Feighelstein et al. [85]
	Behavioral classification.	Adedeji et al. [86]

Other sensor‐based systems	Accelerometers to evaluate activity of growing rabbits.	Mora et al. [92]
	Aerial thermal imaging for group monitoring in free‐range conditions.	Psiroukis et al. [87]

Farm management software	**FarmKeep Rabbit app**: a cloud‐based solution designed for rabbit management, featuring breeding cycle tracking, health monitoring, and financial record‐keeping.	https://www.farmkeep.com/farm-type/rabbit-management
	**Cunitec**: an advanced software for rabbit, chinchilla, and cavy farms, offering over 130 standard reports, mobile data collection, and cloud synchronization.	https://www.agritecsoft.com/cunitec/en/
	**OurRabbitry app**: focuses on pedigree creation, health record tracking, and lineage visualization, simplifying rabbit breeding operations.	https://ourrabbitry.com/
	**Bunny Trails software**: offers multispecies support with features like photos, pedigrees, weights, and show winnings, suitable for various animal breeders.	https://rabbittalk.com/threads/which-software.34327/
	**Rabbitry Farm log management app**: a mobile‐friendly application for tracking breeding, health checks, and financials, accessible on both Android and iOS platforms.	https://play.google.com/store/apps/details?hl=en_US%26id=fi.hassan.rabbitry%26utm
	**Bunny Book**: utilizes OCR technology to scan and upload rabbit pedigrees, facilitating instant transfers and production tracking.	https://bunnybook.org/?utm

*Note:* Bold text indicates the names of the farm management software applications.

Despite the promising advancements in precision feeding for rabbit farming, several challenges and considerations must be addressed to ensure effective implementation and sustainable outcomes. The development and application of precision nutrition techniques face economic and logistical challenges, including the high cost of technology and the need for appropriate infrastructure [93]. There are also conceptual limitations and pitfalls in the development and application of precision nutrition techniques, necessitating careful consideration of the most appropriate and relevant information needed for optimal functioning [89]. Additionally, in intensive rabbit farming systems, strategies such as optimizing fiber requirements and adjusting dietary energy levels, especially in the finishing stage, can reduce feed conversion ratios [90].

## 6. Discussion

The advancement of rabbit production systems requires a shift from conventional, resource‐driven approaches toward welfare‐centered strategies that integrate housing design, nutrition, health management, and technological innovation. Current evidence highlights that enriched and spacious housing not only supports natural behaviors such as digging, jumping, and social interaction but also reduces the incidence of stress‐induced pathologies and aggression [15, 16, 28, 52, 59, 94]. However, the implementation of such systems faces practical challenges, particularly regarding costs, labor intensity, and adaptation to existing farm infrastructures [71, 72]. These constraints underscore the need for gradual but systematic transformation of housing environments in line with welfare principles and consumer expectations [95].

The monitoring of animal welfare through behavioral and physiological indicators is increasingly recognized as a cornerstone of preventive management. The integration of modern welfare assessment methods—including behavioral monitoring, physiological indicators, and environmental measurements—allows for early detection of welfare concerns and informed management decisions. Tools such as the Welfare Quality scoring system provide standardized criteria for evaluating welfare, supporting both research and commercial applications [19]. The adoption of automated systems such as video surveillance and biosensors can significantly enhance accuracy and timeliness in detecting welfare issues [17, 18]. The PLF and digital monitoring technologies represent a promising avenue for continuous, individualized welfare assessment, although their use in rabbit production remains in early development [77, 80]. Nevertheless, the integration of such technologies raises questions of economic feasibility for small‐ and medium‐scale producers, indicating that their widespread use will depend on cost reduction, knowledge transfer, and training [96]. Nutritional management represents another critical determinant of welfare. High‐fiber diets combined with balanced nutrient profiles are essential for gastrointestinal and dental health, yet feed costs and availability of high‐quality roughage often limit farmers′ ability to meet optimal standards [97]. This illustrates the persistent tension between economic efficiency and welfare enhancement, requiring innovative feeding strategies and policy support to align both objectives [98].

Health management and biosecurity remain the foundation of sustainable production, with vaccination, isolation, and hygiene practices reducing both mortality and productivity losses [13]. The prevention of common diseases such as viral hemorrhagic disease, myxomatosis, and coccidiosis is tightly linked to rabbit welfare [42]. Nonetheless, the effectiveness of these measures is strongly influenced by husbandry conditions, particularly microclimatic balance, which is often compromised in intensive settings [16, 52]. This suggests that preventive health strategies cannot be detached from broader environmental and managerial improvements. Another decisive factor is the human dimension of welfare. Farm personnel represent the primary link between welfare principles and their practical application, making staff training in handling, observation, and stress reduction indispensable [99]. Yet, cultivating a welfare culture requires more than technical instruction; it depends on fostering attitudes that recognize welfare as integral to both productivity and ethical responsibility [100].

Finally, regulatory frameworks and certification schemes play a dual role in shaping practice and consumer trust. Although compliance ensures minimum standards, voluntary adoption of higher level certifications can serve as a market differentiator [47].

However, the uneven pace of regulatory development across regions, combined with economic disparities, may result in heterogeneous standards of rabbit welfare worldwide. PLF and smart technologies could mitigate some of these discrepancies by enabling individualized care and data‐driven management, but their long‐term success will depend on balancing technological potential with socioeconomic realities [18, 101]). Table 4 summarizes key measures for enhancing animal welfare in farm settings, alongside their practical implications for management and operational practices.

**Table 4:** Implication for practice.

Category	Key measures	Practical implication
Housing and environmental design	Enriched, spacious housing with platforms, shelters, bedding, and opportunities for natural behaviors. Ensure proper group formation.	Reduces aggression and supports social well‐being
Behavioral and physiological monitoring	Regular observation of behavior, clinical assessments, and physiological indicators (e.g., cortisol, body condition) using integrated automated monitoring technologies including sensors and video analysis.	Early detection of welfare issues; reduces stress and improves management precision
Health management and biosecurity	Implementation of vaccination programs, strict biosecurity protocols, and isolation of sick animals. Maintaining optimal microclimatic conditions.	Prevents infectious disease and reduces stress susceptibility
Staff training and welfare culture	Personnel training in animal handling, early disease detection, behavioral observation, and stress‐minimizing procedures. Promotion welfare culture within the farm.	Enhances compliance with standards and overall care quality
Regulatory compliance and certification	Monitor national and international welfare regulations and pursue certification (e.g., Global G.A.P., Animal Welfare Approved).	Improves market access and demonstrates ethical practices
Technological integration	Precision livestock farming (PLF) and smart monitoring/feeding systems.	Enables individualized care, optimizes management decisions, and facilitates early intervention for welfare concerns

By adopting these integrated practices, farms can ensure the well‐being of rabbits, improve productivity, and meet ethical and market expectations for sustainable and humane animal production.

The future of rabbit farming lies in the proactive integration of welfare principles into production systems. This requires not only technical innovations but also cultural, economic, and regulatory adaptations. A holistic approach integrating housing design, environmental enrichment, nutrition, health management, behavioral monitoring, and staff training is required. Investments in welfare‐friendly practices not only fulfill ethical obligations but also contribute to sustainable production, improved animal health, and enhanced product quality.

## 7. Conclusion

Ensuring the welfare of rabbits in industrial farming is critical for aligning ethical animal husbandry with the demands of sustainable agricultural production. This review emphasizes the multifaceted nature of rabbit welfare, covering physiological health, psychological well‐being, and the ability to express natural behaviors, guided by the internationally recognized “five freedoms” and Welfare Quality frameworks. The article highlights novel findings that conventional cage systems significantly restrict rabbit behavior, leading to stress‐related disorders, muscle atrophy, and increased disease incidence. Conversely, alternative housing models such as enriched enclosures and group housing promote physical activity, social interaction, and psychological health, reducing stereotypies and aggression.

The assessment of rabbit welfare requires a comprehensive approach that integrates behavioral observation, physiological indicators, clinical examinations, environmental monitoring, and standardized welfare scoring systems, such as Welfare Quality. Modern digital technologies, including PLF, automated monitoring, and machine learning applications, offer promising tools for continuous welfare assessment and evidence‐based management, though their implementation in rabbit farming is still in the early stages.

The review further identifies the pivotal role of qualified farm personnel in practical welfare implementation, emphasizing training in animal handling and early disease recognition to enhance care quality. Integrating welfare‐centered housing, nutrition optimized for gastrointestinal health, rigorous biosecurity, and advanced monitoring systems offers a holistic approach to improving rabbit welfare while maintaining economic viability.

Ultimately, these findings reflect a necessary shift from productivity‐driven paradigms toward welfare‐focused strategies that benefit animals, producers, and consumers alike. Continued research, policy development, and knowledge transfer are crucial to promote widespread adoption of these innovations and establish higher global standards for rabbit farming.

## Funding

No funding was received for this manuscript.

## Conflicts of Interest

The authors declare no conflicts of interest.

## Data Availability

The data that support the findings of this study are available from the corresponding author upon reasonable request.
